# Hemodynamic Performance of Multilayer Stents in the Treatment of Aneurysms with a Branch Attached

**DOI:** 10.1038/s41598-019-46714-7

**Published:** 2019-07-15

**Authors:** Zhongyou Li, Lijuan Hu, Chong Chen, Zhenze Wang, Zhihong Zhou, Yu Chen

**Affiliations:** 1 Department of Applied Mechanics, Sichuan University, Chengdu, 610065 China; 2 Third Department of Internal Medicine, Friendship Hospital, Xi’an, 710072 China; 3 College of Manufacturing Science & Engineering, Sichuan University, Chengdu, 610065 China; 4 Key Laboratory of Rehabilitation Aids Technology and System of the Ministry of Civil Affairs & Beijing Key Laboratory of Rehabilitation Technical Aids for Old-Age Disability, National Research Center for Rehabilitation Technical Aids, Beijing, 100176 China

**Keywords:** Experimental models of disease, Biological models

## Abstract

Although multilayer stents (MSs) can be used to treat aneurysm effectively, for some aneurysms with branches attached, the hemodynamic mechanisms are still unclear. In this work, we modeled five cases that involve 1–4-layer stents implanted in aneurysms with side branches, and the numerical approach was used. Case 1 corresponds to an aneurysm without a stent, and cases 2–5 represent 1–4-layer stents being employed within aneurysms, respectively. The results showed that the velocity within the sac declined dramatically and the eddies’ intensity weakened with increased number of stent layers, time-averaged wall shear stress (TAWSS), and nitric oxide production rate (TAR_NO_) dropped linearly with increase in stent porosity, and oscillatory shear index (OSI) and relative residence time (RRT) increased evidently with MS intervention. Moreover, the MSs had a slight effect on the patency of the side branch; its flow rate was still above the normal case than without aneurysm. It can be concluded that MSs are helpful in promoting the growth of thrombus within the aneurysm through an isolated hemodynamic environment and keeping the branch unobstructed, but more clinical evidences are required.

## Introduction

An aneurysm is a life-threatening angiopathy (mortality = 70–95%)^1,2^, resulting from local dilation of blood vessels. The risk of aneurysmal rupture increases with the growth in its size, and massive hemorrhage is the most dangerous outcome after rupture, so it is very risky not to intervene early. The elderly constitute a high-risk group suffering from aneurysm^3^, which is mainly dominated by vascular aging, and thus there will be more challenges as the world’s population ages.

The stent graft has been developed as a feasible approach for aneurysm treatment^4–7^. The principle is the isolation of the aneurysm from circulation to decrease the pressure inside the sac and promote the formation of thrombus, but there are some limitations, for instance, for aneurysms with branches attached. Given the patency of the branch, the stent graft is inapplicable because important branches, such as the renal artery, mesenteric artery, and hepatic artery, are blocked while the aneurysm is excluded. Although the chimney^8–11^ and fenestrated techniques^12–15^ are commonly used in the management of aneurysms with visceral vessels, these stents usually require customization because of individual differences, and the process of surgery is difficult and intricate for clinicians in terms of precise deployment. Therefore, a more convenient scheme is required. Recently, multilayer stents (MS, two or more bare-metal stents overlapping with each other) have been gradually applied to treat aneurysms, and the curative effect has been proven in terms of both clinical follow-up trials and hemodynamic theories. A retrospective study including 61 patients exhibiting complex aneurysms was conducted by Ibrahim *et al*.^16^, wherein the MS technical success was 95%, and freedom from rupture and reintervention were 97.5% and 100%, 96% and 84%, and 86% and 75% for 1 month, 6 months, and 12 months, respectively. With the MS isolation, shrinkage occurred with the growth of sac thrombosis^17,18^, and it was even found that maximum shrinkage could be as high as 57%^18^; the hemodynamics indicated that the blood flow and pressure inside the sac declined immediately after MSs were employed^17^. Furthermore, a numerical investigation^19^ concluded that the pressure on the aneurysmal wall distributed more uniformly, wall shear stress (WSS) declined, and oscillatory shear index (OSI) and relative residence time (RRT) increased with two-layer stent employment.

However, although the significance of MSs has been recognized, for some aneurysms that involve side branches, the hemodynamic mechanisms are doubtful, and the optimal porosity of MSs is still unclear. As the risk of aneurysm rupture decreases with improvement in the stents’ isolation, the blood supply of the side branch could be blocked when the stents’ porosity is denser. Thus, there is a contradiction between isolation and patency of the visceral vessel with the increase in the number of MS layers. Consequently, a numerical study is required before clinical practice to determine the optimal number of layers in the stents for improved isolation and keeping the branch unobstructed. In the present study, we modeled 1–4-layer stents, besides conventional parameters related to thrombosis such as WSS, OSI, and RRT; the nitric oxide (NO) production rate in the endothelium was also taken into account.

## Materials and Methods

### Geometries and meshes

**Figure 1:**
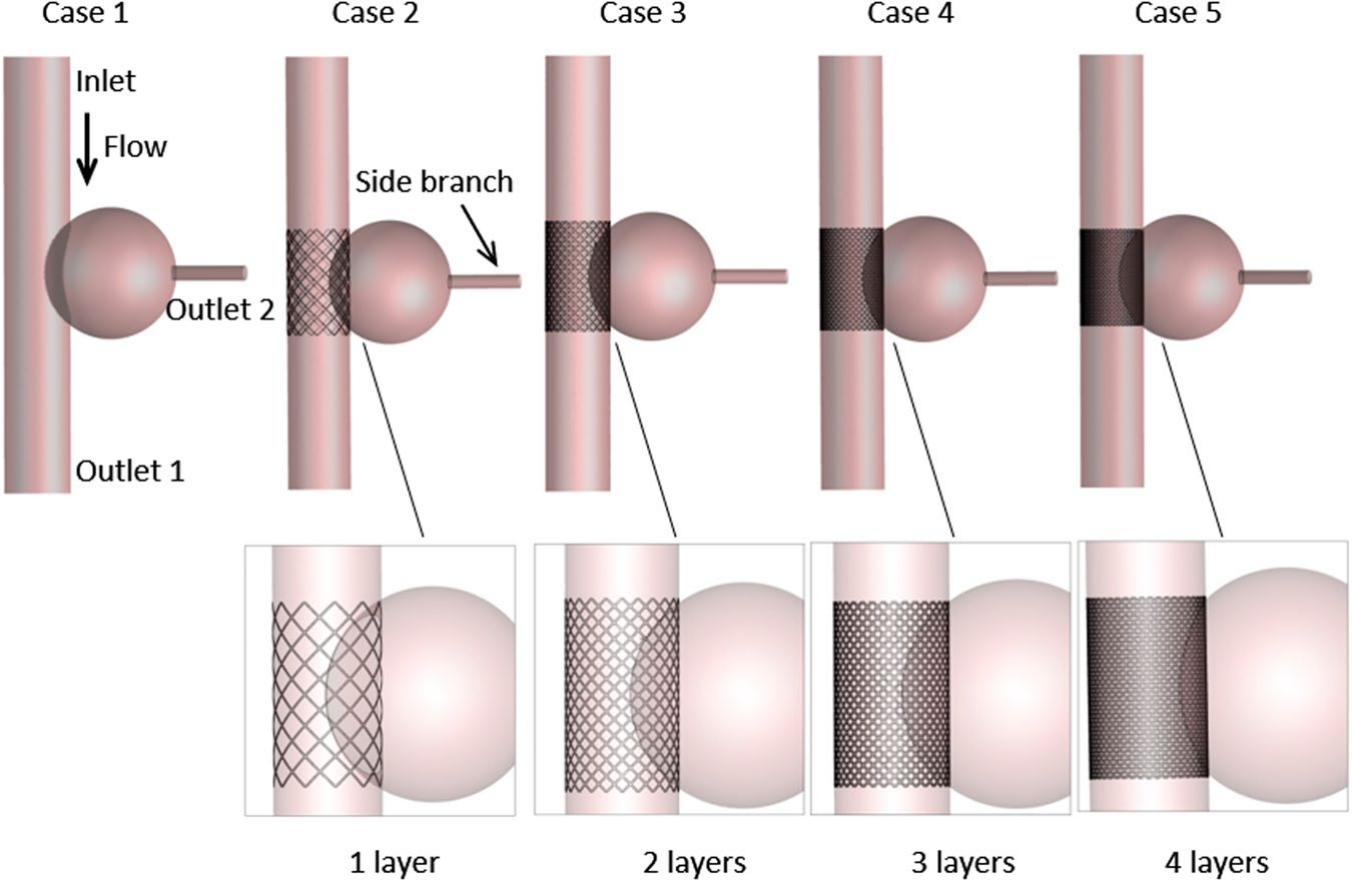
Configuration of an aneurysm with a side branch. Case 1 represents the aneurysm with no stent; Cases 2–5 correspond to aneurysms intervened by 1–4-layer stents. The thickness of the square elements of the stent is 0.1 mm. The positions of three boundaries are shown, including an inlet and two outlets.

We constructed five three-dimensional models (Fig. 1) for simulation using SolidWorks (version 16.0, Dassaut Systemes S.A, USA). A saccular-type aneurysm was selected and we ignored the thrombus that already existed inside the aneurysm because of sophisticated construction and pronounced individual differences^20^. We aimed to compare the differences between these models qualitatively, and we believed that there would be no effects on the results and conclusions of this study.

In the present study, case 1 represents abdominal aorta aneurysm (AAA) without stents, and cases 2–5 correspond to AAAs with 1–4-layer stents employed (According to Brent’s patent, the stents’ porosities are 85.2%, 72.3%, 60.5%, and 52.4%, respectively, for 1–4-layer stents, and the thickness of struts is 0.1 mm; the configuration of the stent element is square.), respectively. A side branch was attached to the aneurysm, and its diameter (4 mm) was set the same as that of the kidney artery.

The meshes of five cases were automatically produced from ICEM CFD (version 16.0, ANSYS Inc., USA); tetrahedral-type meshes were used in the whole region of the models, but some sensitive regions, such as stents and arterial wall, were refined. The command “grid adaption” in FLUENT (version 16.0, ANSYS Inc., USA) was used for keeping the mesh independent; when the results were irrelevant to the number of meshes, it stopped refining.

### Governing equations

**t**he time-dependent, incompressible, and three-dimensional Navier-stokes equation was used in this solution, including mass conservation and momentum conservation equations as follows:

1 $$\nabla \cdot u=0$$

2 $$\rho (\frac{\partial u}{\partial t}+u\cdot \nabla u)=-\nabla P+\mu {\nabla }^{2}u$$

where *u* and *P* represent the velocity and pressure vector, respectively. *ρ* (1050 kg/m^3^) and *μ* (0.0035 Pa·s) denote the density and dynamic viscosity of blood^21,22^, respectively.

### Boundary condition

**Figure 2:**
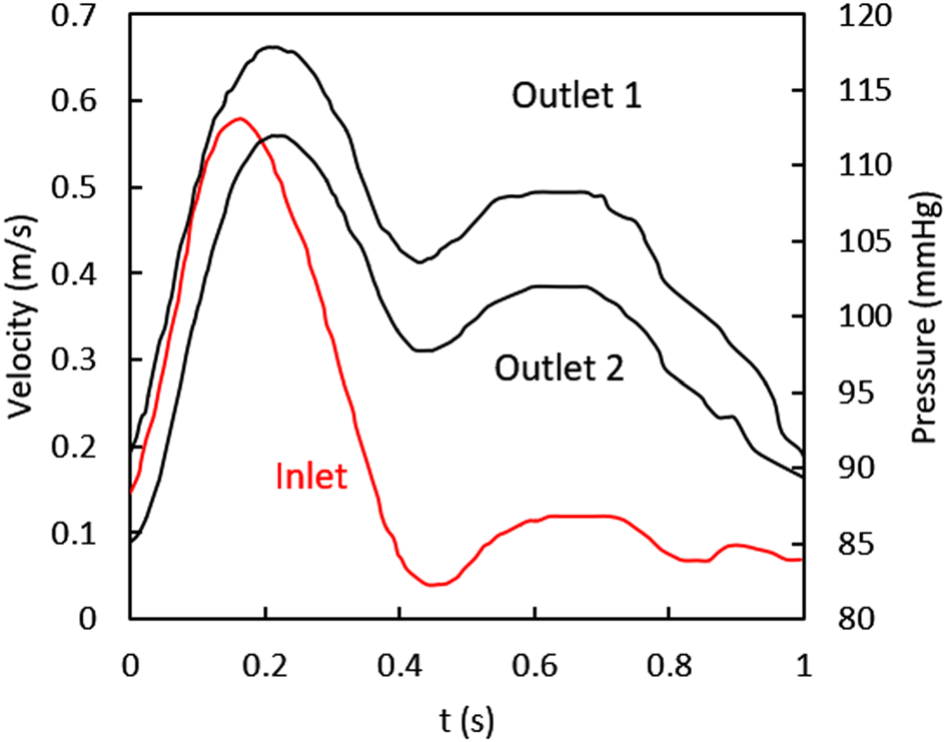
Boundary condition for velocity inlet and two pressure outlets.

According to the literature^23^, a pulsating flow (the mean Reynolds number equals to 1296, which is less than the critical value of turbulence flow) was imposed on the aorta inlet (Fig. 2), and different pulsating pressure boundary conditions were given for two outlets. Because the diameter of the side branch is smaller than that of the aorta, the pressure of outlet 2 is slightly lower than that of outlet 1 (Fig. 2). The arterial wall, aneurysmal wall, and stents were assumed to be rigid with no-slip boundary condition in all cases^24^.

### Numerical solution

Computational domains were solved via commercial software FLUENT (version 16.0, ANSYS Inc., USA), which is based on the finite volume method. The SIMPLE solution was selected for pressure-velocity coupling. Least-Squares Cell-Based, Standard, and Second-Order Upwind schemes were utilized for the discrete form of gradient, pressure, and momentum, respectively. Four cardiac cycles were calculated for all cases to keep the solution stable, and the data of the fourth cardiac cycle was extracted for analysis^19^.

### Hemodynamic parameters

The average WSS of each wall cell over the entire cardiac cycle was expressed by the time-averaged wall shear stress (TAWSS); the formula for the same can be written as

3 $$TAWSS=\frac{1}{t}{\int }_{0}^{t}|WSS(c,t)|\cdot dt$$

where t and c are the cardiac cycle period and cell on the arterial wall, respectively.

OSI was introduced for evaluating the directional change in WSS during the cardiac cycle, defined as:

4 $$OSI=\frac{1}{2}[1-(\frac{|{\int }_{0}^{t}WSS(c,t)\cdot dt|}{{\int }_{0}^{t}|WSS(c,t)|\cdot dt})]$$

Therefore, the value of OSI ranges from 0 to 0.5, where 0 indicates WSS without oscillation, and 0.5 indicates WSS with an instantaneous frequent deviation^25^.

RRT represents the residence time of blood near the arterial wall and is a function of TAWSS and OSI, expressed as^25^

5 $$RRT=\frac{1}{(1-2\cdot OSI)\cdot TAWSS}$$

Besides TAWSS, OSI, and RRT, we also took the NO production rate (R_NO_) of the endothelium into consideration, according to the correlation between R_NO_ and WSS by experimental measurement^26,27^:

6 $${R}_{NO}(t)={R}_{{\rm{basal}}}+{R}_{\max }\cdot \frac{|WSS(c,t)|}{|WSS(c,t)|+\alpha }$$

where R_basal_ = 2.13 nM s^−1^, R_max_ = 457.5 nM s^−1^, and α = 3.5 Pa. Time-averaged R_NO_ (TAR_NO_) can be calculated as

7 $$TA{R}_{NO}=\frac{1}{t}{\int }_{0}^{t}{R}_{NO}(t)\cdot dt=2.13+457.5\cdot \frac{1}{t}{\int }_{0}^{t}(\frac{|WSS(c,t)|}{|WSS(c,t)|+3.5})\cdot dt$$

## Results

### Flow pattern

**Figure 3:**
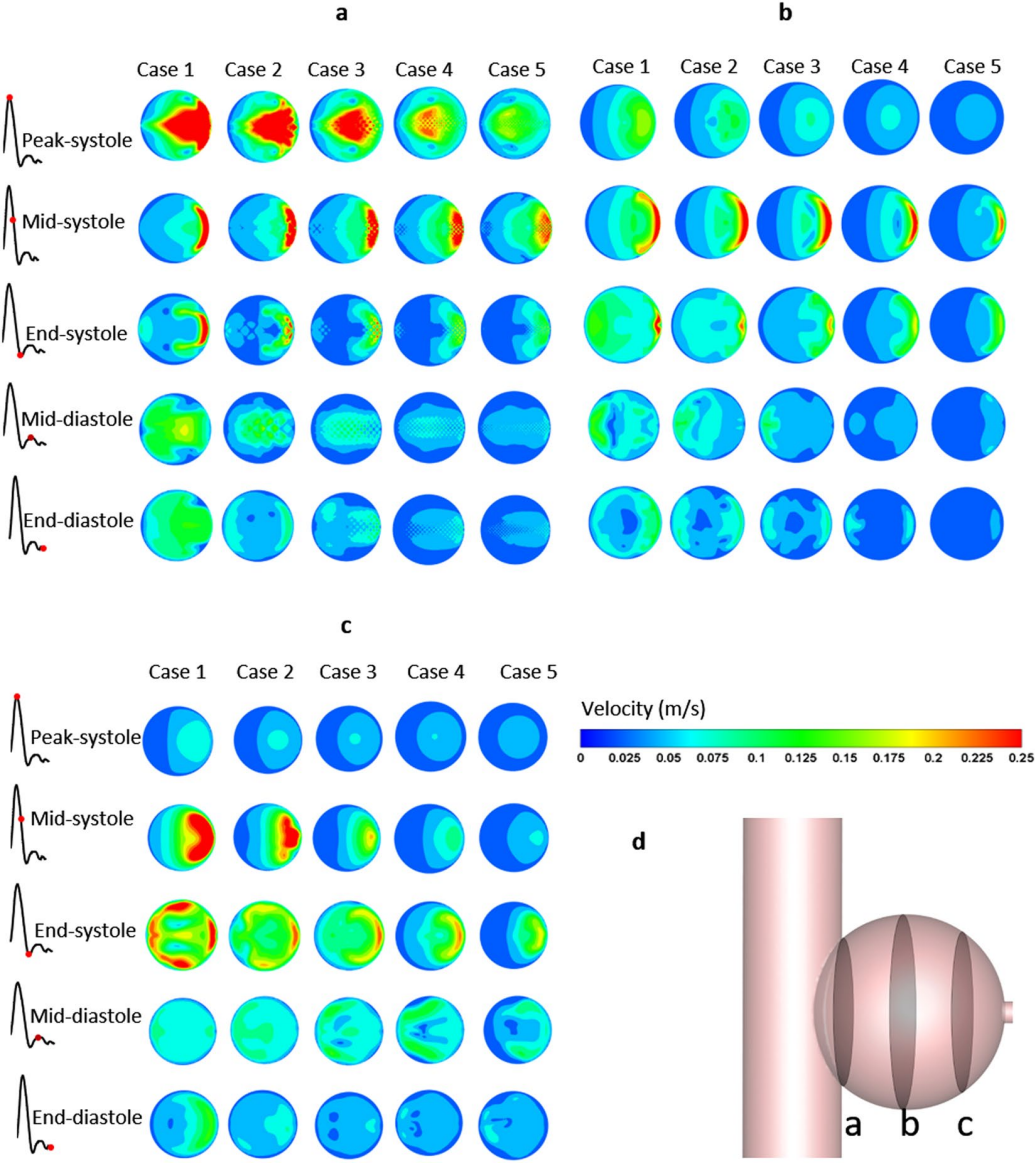
Velocity profile during cardiac cycle. (**a–c**) Represent the regions near the stents, middle aneurysm, and near the side branch, respectively; f indicates the position of slices (**a–c**).

**Figure 4:**
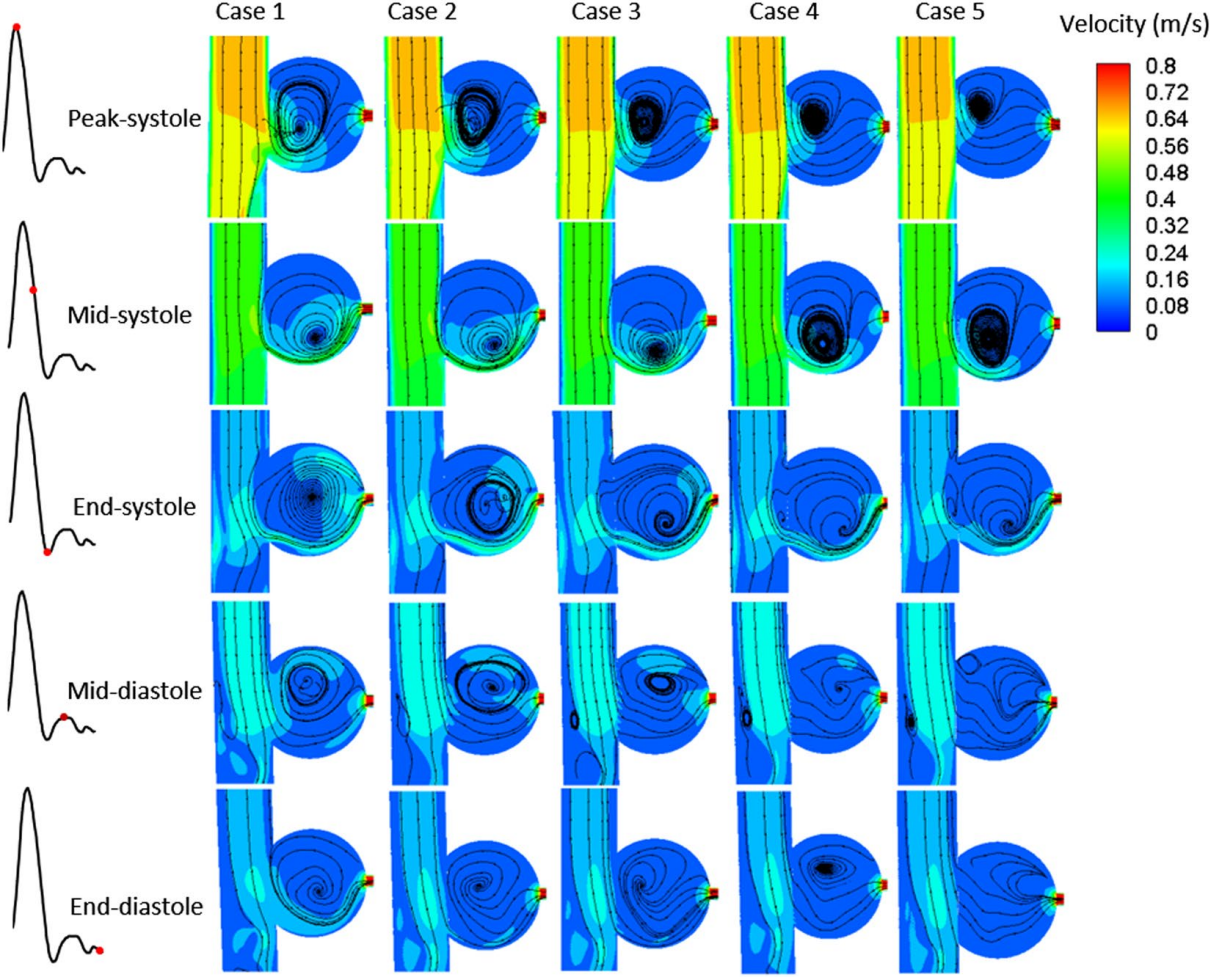
Evolution of streamlines and eddies with different stent interventions.

Significant differences are observed between different points of the cardiac cycle (Figs 3 and 4), owing to inertia and blood flow into the sac mainly through the downstream of the aneurysm in the systole, but in the middle diastole, high-speed blood flow occurs in the upstream of the aneurysm. Although the inlet velocity declines from peak systole to end systole, it is not the same in the aneurysm, such as profile c (Fig. 3) near the side branch; it is interesting that peak velocity occurs in the middle and end systole near this region, but relatively low flow occurs in the peak systole. With stent intervention, besides the decline in blood flow in the aneurysmal sac, chaotic flow is observed in the region near the stents (Fig. 3, profile a) and disappears at profiles b and c, and the isolation effects improve with the increase in the number of stent layers. However, the obvious change can be found after stents with more than two layers are employed, and especially for four-layer stent implants, the flow becomes more uniform and the peak velocity in the downstream vanishes, except for the middle systole.

Eddies are usually relevant to saccular-type aneurysms, and the position of the eddy center varies with the cardiac cycle (Fig. 4). The eddy center moves according to the movement of peak velocity of the aneurysm; therefore, the eddy occurs at the aneurysmal downstream and upstream in the middle systole and middle diastole, respectively. Stents plays a significant role in affecting the position of eddies, but a few obvious laws can be observed. However, owing to the eddy results from the difference in velocity between adjacent regions, the diameter and intensity decrease with MS blocking, as shown in Fig. 4, especially in the diastole in which the eddy is going to annihilate.

### Pressure

**Figure 5:**
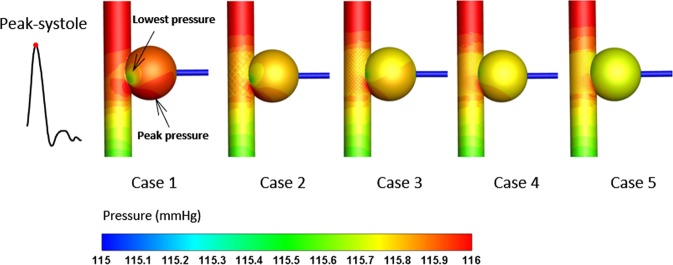
Distribution of aneurysmal pressure in the peak systole. The peak and lowest pressure in the downstream of the aneurysm are indicated by arrows.

Pressure is a key factor in aneurysmal growth and rupture, and the risk increases as the pressure rises; therefore, pressure control is crucial for keeping the aneurysm stable. Generally, owing to maximum blood pressure in the systole, it is necessary to take this period into consideration (Fig. 5). The peak pressure occurs in the bottom of the sac where the velocity is the highest, while the lowest pressure can be found at the center region of the eddy. Although minimal change can be observed after stent employment, peak pressure gradually vanishes and the pressure distributes more uniformly with increase in the number of stent layers.

### TAWSS, OSI, and RRT

**Figure 6:**
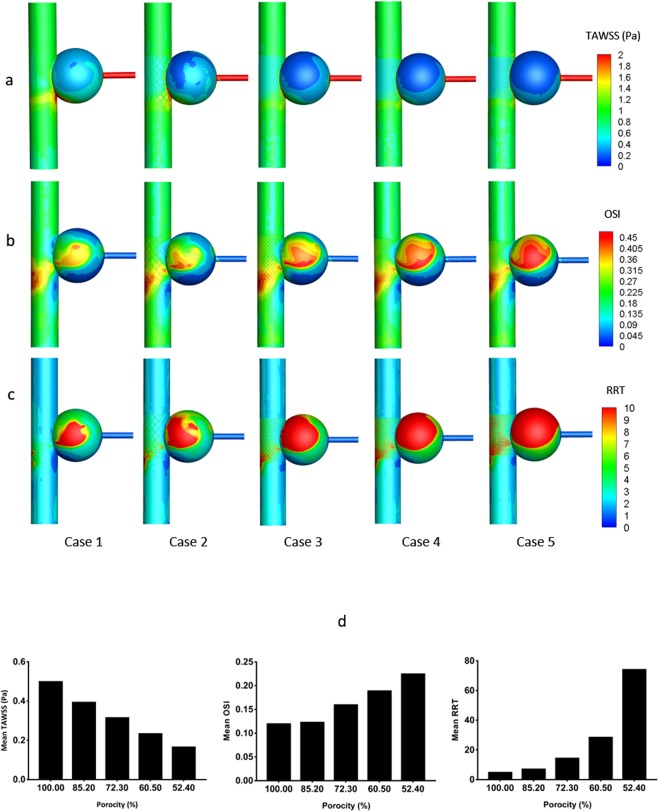
TAWSS, OSI, and RRT on the aneurysmal wall. (**a–c**) Correspond to the TAWSS, OSI, and RRT contours of five cases; d refers to the mean values of TAWSS, OSI, and RTT between different stents’ densities in the bar graphs.

TAWSS, OSI, and RRT are the main parameters related to vasculopathy, such as for thrombosis; both quantitative results and contours are shown in Fig. 6. TAWSS on the aneurysmal wall evidently declines with stent employment (Fig. 6a), and the relatively low TAWSS region occurs at the center of the sac wall; the denser the MSs, the lower is the TAWSS on the aneurysmal wall. The bar graph shows that the mean TAWSS decreases with stent porosity linearly (Fig. 6d), and it decreases by 0.07 Pa for every 10% reduction in stent porosity. Clearly, both OSI (Fig. 6b) and RRT (Fig. 6c) on the sac wall rise with increasing number of stent layers. According to the bar graphs (Fig. 6d), the growth rates are 3%, 33.7%, 58.2%, and 88.5% and 47.4%, 206.5%, 512.4%, and 1501.4%, for the mean OSI and RRT on the sac wall, respectively, whereas the mean RRT increases more dramatically, especially for the region of the aneurysmal center wall.

### Nitric Oxide

**Figure 7:**
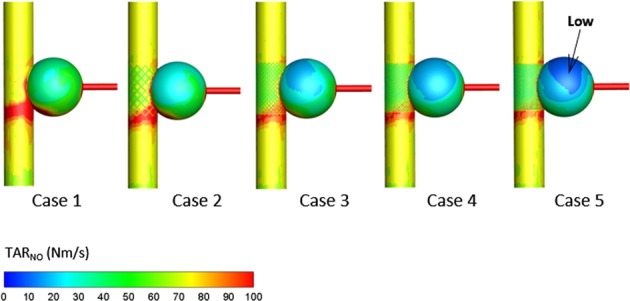
Nitric oxide production rate (TAR_NO_) in aneurysmal endothelium. Low TAR_NO_ is indicated by an arrow.

**Figure 8:**
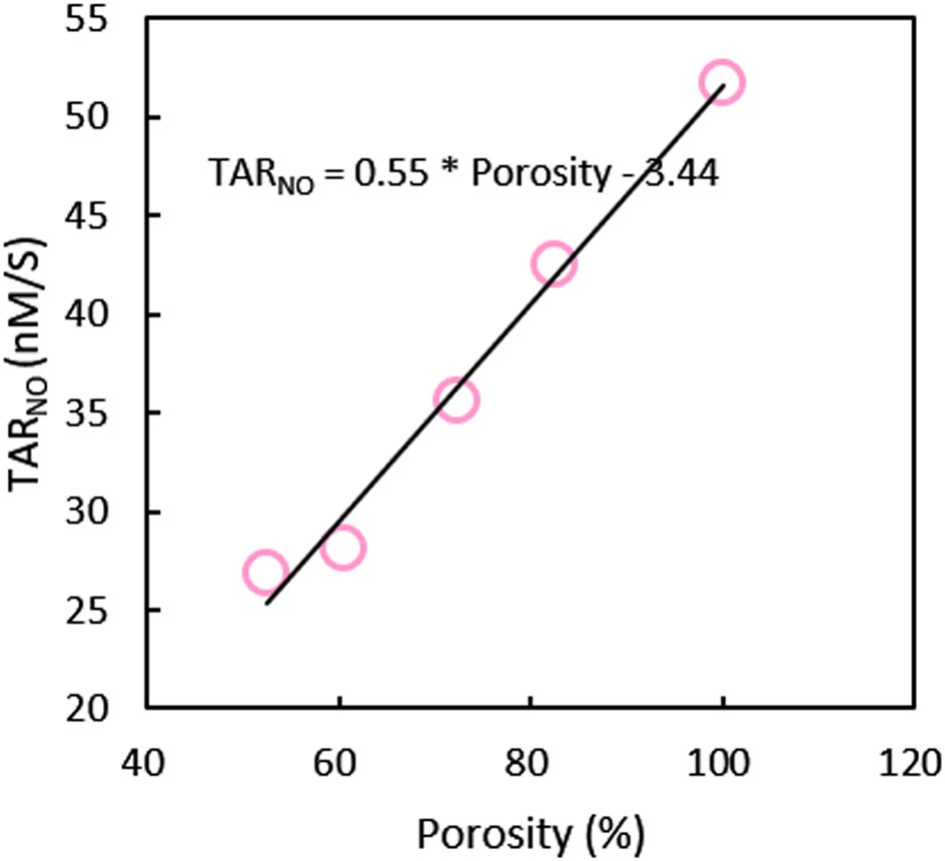
Relationship between stent density and mean TAR_NO_ on the aneurysmal wall.

NO, produced in the endothelium by shear stress stimulating, plays a significant role in vascular function regulation. The time-averaged NO production rate (TAR_NO_) is shown by the contour and quantitative data in Figs 7 and 8, respectively. Obviously, the low TAR_NO_ occurs at the center region of the sac wall, which is the same as TAWSS (Fig. 6). As the number of stents is increased, the TAR_NO_ on the sac wall reduces. Figure 8 shows the linear relationship between stent density and mean TAR_NO_; the mean TAR_NO_ decreases by 5.5 nM·s^−1^ for every 10% reduction in stent porosity.

### Patency of side branch

**Figure 9:**
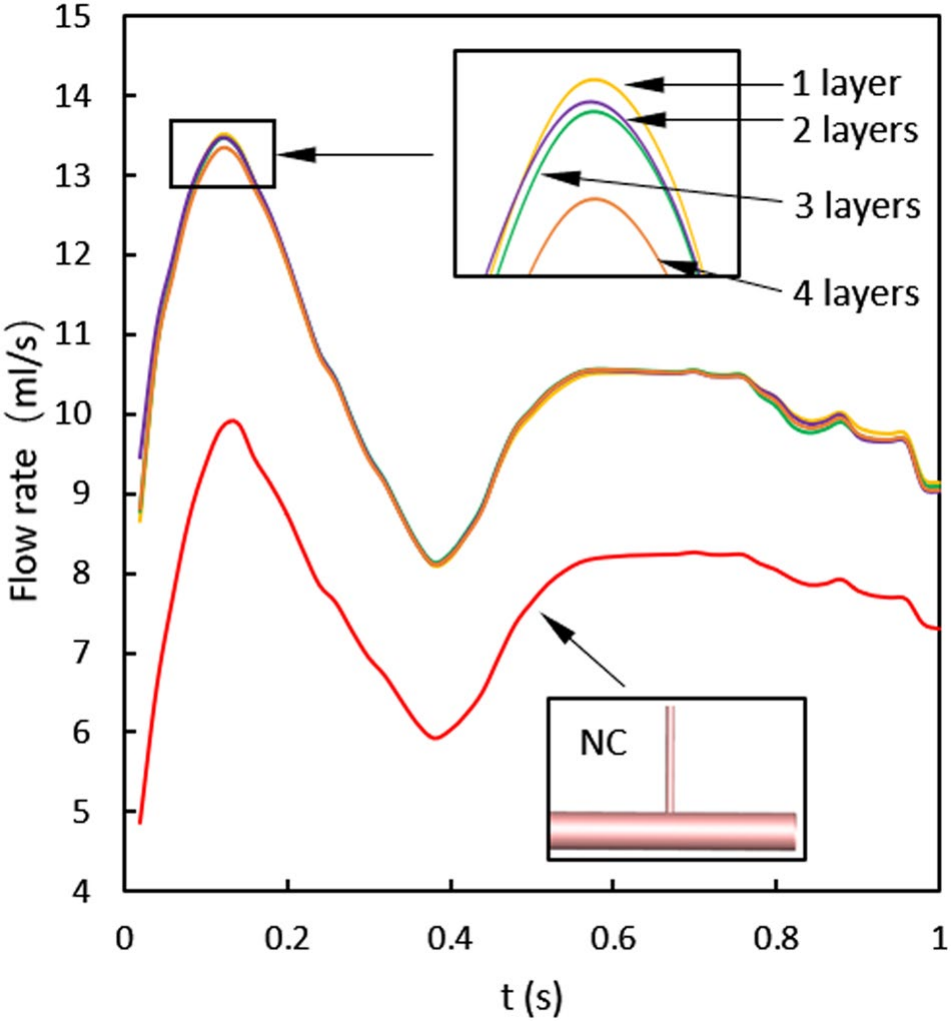
Flow rate of the side branch during the cardiac cycle. NC: normal case without aneurysm.

Supervising the flow rate of the side branch is crucial for the evaluation of its patency. The variation in the volume flow rate of outlet 2 with the cardiac cycle is shown in Fig. 9. For a fair comparison, the normal case without aneurysm was considered. Clearly, although a slight difference is observed between 1–4-layer stents and the flow rate reduces with the increase in the number of stent layers, they are still above the normal case, and thus there is no doubt that the visceral vessel is unobstructed.

## Discussion

Although both the numerical investigations^19^ and follow-up trials^16–18^ confirmed that bare-metal stents overlapping with each other could treat aneurysms effectively, for some aneurysms that involve side branches, the hemodynamic mechanisms are doubtful. In the present study, the differences after 1-4-layer stenting within aneurysms were explored in terms of pulsatile flow field, TAWSS, OSI, RRT, and TAR_NO._

The results of the flow field show that MSs play a substantial role in regulating the blood in the aneurysmal sac, and the velocity declines with increasing number of stents. Owing to inertia, the peak flow occurred on the bottom region of the aneurysm where the pressure is slightly higher correspondingly. Despite the low velocity corresponding to the high pressure, a slightly higher pressure was observed on the wall of the bottom region of the aneurysm than at the top, which can be interpreted as being due to the following reasons: firstly, because of blood flow inertia and saccate shape of aneurysm, the eddy was formed inside the sac, which is consistent with previous numerical studies^28,29^, causing blood to flow from the aneurysmal bottom to the top. Consequently, since blood flow is always driven by pressure drop in the circulation system, there is exactly a pressure drop between the bottom and top of the aneurysm; secondly, according to the law of energy conservation, as the friction loss from the bottom of the aneurysm to the top is slightly greater than the reduction in kinetic energy loss, there is a slightly higher pressure on the bottom of the sac than on the top. However, the velocity profile became uniform after four-layer stent implantation, which is the reason why the pressure on the sac wall becomes more uniform. Therefore, to some extent, with devices employed, the improvement in the hemodynamic environment helps decrease the risk of stress concentration caused by the uneven distribution of pressure. In addition, eddies are the result of local rotations caused by flow differences, and great differences in velocity were observed between the bottom and top of the aneurysm; hence, a saccular-type sac is always accompanied by rotary flow. With stents blocking the blood flow, the intensity of rotation becomes weak because of uniform velocity, which may help the aneurysm to freedom from scour^20^ and keep it stable.

Our analysis of CDF results demonstrated that the TWASS on the sac wall declined with stent layers, which is strongly relevant in decreasing the velocity^25,30^. On the contrary, the aneurysmal OSI and RRT became greater after stenting, and the denser the MSs were, the greater were the OSI and RRT. The region of low TAWSS, high OSI, and high RRT occurs at the centers of the eddies, because the closer the position is to the rotating center point, the slower the velocity will be. The combination of the low TAWSS, high OSI, and high RRT is an enabling environment for thrombus growth^19,25^, especially for high RRT, which would promote platelet aggregation to thrombose on the aneurysmal wall^19^. According to the analysis, we predicted that the thrombus would grow near the region of the center sac wall first, and the four-layer stent with a density of 52.4% has the best isolation when compared with other cases, which supports the view that the optimal regulation of the hemodynamic environment can be achieved by MSs with a porosity of 50–70%^25,31^.

NO produced from vascular endothelium is a biological signal used to regulate the arterial wall function^32,33^, and it plays an important role in angiopathy. According to experimental measurements, there is a strong association between NO generation rate and WSS^26,27^. Our study indicated that the TAR_NO_ on the aneurysmal wall linearly declined with the stents’ porosity reduction, which is similar to the result of TAWSS. Hence, these results suggest that the MSs severely inhibit the production of NO and would aggravate the intracapsular thrombosis.

The patency has been confirmed in follow-up trails^16^, but the studies did not consider the impact of the number of layers, and the hemodynamic mechanism is still insufficiently understood. In this study, a comparison of flow rate between MSs and the normal case was carried out with the objective of providing theoretical evidence of the branch’s patency. Our results revealed that the time-varying flow rate of the side branch is still above the normal case with no aneurysm in all stents’ cases (cases 2–4) because the resistance reduced with the dilation of the arterial wall. Moreover, as opposed to the vascular resistance, the stents have much less resistance because of the extremely low thickness. Therefore, a four-layer stent, at the least, can be used in the treatment of aneurysm for better isolation, and there were no concerns related to the patency of the branch.

### Limitation of this study

This work still has some limitations. Clinical and *in vitro* experiments are required to validate our results, and we will take this part of the work into consideration in the future. Currently, we aim to evaluate the feasibility of multilayer stents in terms of hemodynamics via the numerical approach. Because of individual differences, we did not consider the effect of the angle of the side branch on the results, but we believe that the conclusion would be consistent. The size of the aneurysm greatly affects the resistance of the side branch, and hence the isolation and patency would be different, but the relative results between cases 1–5 would not be changed. We will consider the size effects in future work.

## Conclusion

The method of overlapping stents can isolate the aneurysm effectively and keep the branch unobstructed. Especially, the isolation effect improves with increased number of stent layers. The mechanisms and performances indicate that TAWSS and TAR_NO_ on the aneurysmal wall decline and OSI and RRT increase with MS employment, which is helpful in promoting the growth of thrombus within the sac.
